# Stevia Prevents Acute and Chronic Liver Injury Induced by Carbon Tetrachloride by Blocking Oxidative Stress through Nrf2 Upregulation

**DOI:** 10.1155/2018/3823426

**Published:** 2018-04-19

**Authors:** Erika Ramos-Tovar, Erika Hernández-Aquino, Sael Casas-Grajales, Laura D. Buendia-Montaño, Silvia Galindo-Gómez, Javier Camacho, Víctor Tsutsumi, Pablo Muriel

**Affiliations:** ^1^Laboratory of Experimental Hepatology, Department of Pharmacology, CINVESTAV-IPN, Apartado Postal 14-740 Mexico City, Mexico; ^2^Department of Infectomics and Molecular Pathogenesis, CINVESTAV-IPN, Apartado Postal 14-740 Mexico City, Mexico; ^3^Department of Pharmacology, Apartado Postal, CINVESTAV-IPN, 14-740 Mexico City, Mexico

## Abstract

The effect of stevia on liver cirrhosis has not been previously investigated. In the present study, the antioxidant and anti-inflammatory properties of stevia leaves were studied in male Wistar rats with carbon tetrachloride- (CCl_4_-) induced acute and chronic liver damage. Acute and chronic liver damage induced oxidative stress, necrosis, and cholestasis, which were significantly ameliorated by stevia. Chronic CCl_4_ treatment resulted in liver cirrhosis, as evidenced by nodules of hepatocytes surrounded by thick bands of collagen and distortion of the hepatic architecture, and stevia significantly prevented these alterations. Subsequently, the underlying mechanism of action of the plant was analyzed. Our study for the first time shows that stevia upregulated Nrf2, thereby counteracting oxidative stress, and prevented necrosis and cholestasis through modulation of the main proinflammatory cytokines via NF-*κ*B inhibition. These multitarget mechanisms led to the prevention of experimental cirrhosis. Given the reasonable safety profile of stevia, our results indicated that it may be useful for the clinical treatment of acute and chronic liver diseases.

## 1. Introduction

*Stevia rebaudiana* is a small perennial shrub that belongs to the aster or chrysanthemum family and grows in the Amambay mountain range of Paraguay [1]. Stevia leaves contain specific substances (glycosides) that have a sweet taste without any caloric value [2]. Stevia has been used by South Americans for the treatment of diabetes for many years [3, 4]. Several compounds with beneficial health properties have been reported in stevia leaves [2]. Recently, a comprehensive profile of the compounds present in *S. rebaudiana* leaves was achieved: a total of 89 compounds were identified in the polar and nonpolar extracts of the stevia plant and classified into different families [5]. Although the antihyperglycemic, insulinotropic, and glucagonostatic actions of stevia have been widely studied in diabetic rats [6–9], the potential beneficial effects of this plant on liver diseases [2] have not been previously investigated. In the present study, the *in vivo* antioxidant and anti-inflammatory properties of stevia leaves were studied against acute and chronic liver damage induced by carbon tetrachloride (CCl_4_) administration in rats [10]. This study has reported the first indication that stevia leaves possess strong activity against CCl_4_-induced liver damage through a multitarget mechanism that includes the improvement of antioxidant defense mediated by Nrf2 and blockage of the proinflammatory factor NF-*κ*B.

## 2. Materials and Methods

### 2.1. *S. rebaudiana* Characteristics

In this study, we utilized *S. rebaudiana* Bertoni variety Morita II (stevia), which is commercially procured as Mayan Sweet Stevia® (Yucatan, Mexico). This product is certified by the U.S. Department of Agriculture (USDA). Irrigation was conducted by drip, the pH of the water was 7.5, the pH of stevia was 7.0, and the pH of stony soil was 7.5. Stevia was grown at an altitude of 550 m at 26°C and 60% humidity. The components present in this variety of stevia have been extensively described by several researchers [2, 11–15]; a summary of the main components present in the leaves is shown in Table 1. For administration purposes, stevia leaves were sprayed in the mill with a 1 mm mesh and stored in amber glass bottles to protect them from sunlight before use.

### 2.2. Acute Liver Damage

A dose-response study of the effect of stevia on CCl_4_ acute liver injury was performed in male Wistar rats (200–250 g), and the most effective dose was identified as 100 mg/kg (data not shown). Accordingly, further acute and chronic treatments were performed at this dose. The animals were divided into four groups of eight rats each. The control group received 0.3% carboxymethyl cellulose ((CMC); stevia vehicle) at a dose of 1 mL daily p.o. for 1 week. In the CCl_4_ treatment group, acute liver toxicity was produced by one dose of CCl_4_ (4 g/kg) dissolved in mineral oil and administered through an intragastric tube, as previously described [10, 16]. The CCl_4_ + stevia treatment group was administered with one dose of CCl_4_ (similar to the CCl_4_ group) plus 100 mg of stevia/kg body weight (suspended in 0.3% CMC) daily at 9 am for 1 week through an intragastric tube. The stevia group was administered with 100 mg of stevia/kg body weight daily at 9 am, also for 1 week through an intragastric tube. CCl_4_ was obtained from J.T. Backer (Xalostoc, Mexico State, Mexico).

### 2.3. Chronic Liver Damage

Male Wistar rats (initial weight, 100–120 g) were randomly divided into four groups of 8 rats each. The control group was administered with 0.3% CMC at a dose of 1 mL daily p.o. The CCl_4_ group was administered with 400 mg CCl_4_/kg body weight i.p. 3 times per week, dissolved in mineral oil, Monday, Wednesday, and Friday at 9 am for 12 weeks, as described previously [10]. The CCl_4_ + stevia group was administered with CCl_4_, as in the CCl_4_ group, plus 100 mg of stevia/kg body weight daily at 9 am for 12 weeks through an intragastric tube. The stevia only group was administered with 100 mg of stevia/kg body weight daily at 9 am, also for 12 weeks through an intragastric tube.

The animals were given free access to food (Labdiet® number 5053, Indiana, USA) and drinking water. Body weight gain was assessed once per week. The rats were anesthetized with ketamine and xylazine and then euthanized by exsanguination. Blood was collected by cardiac puncture and centrifuged in tubes at 3000 rpm (12000*g*). The liver of each rat was rapidly removed, weighed, and stored at −75°C. The animals were treated in accordance with Mexican official regulation (NOM-062-ZOO-1999) and technical specifications for the production, care, and handling of laboratory animals, as well as in accordance with the Guide for the Care and Use of Laboratory Animals (NRC, 2011).

### 2.4. Biochemical Analyses

Plasma was obtained for the analysis of alanine aminotransferase (ALT) [17], alkaline phosphatase (AP) [18], gamma-glutamyl transpeptidase (*γ*-GTP) [19], and bilirubin using a commercial kit supplied by Spinreact® (catalog number 1001044, Girona, Spain). Reduced glutathione (GSH) determination was performed in accordance with the method of Sedlak and Lindsay [20]. This reaction involved the oxidation of GSH by the sulfhydryl reagent 5,5′-dithio-bis (2-nitrobenzoic acid) (DTNB) to form the yellow derivative 5-thio-2-nitrobenzoic acid (TNB), with the absorbance measured at 412 nm. The glycogen content was quantified by using the anthrone method, with the absorbance measured at 620 nm [21]. The extent of lipid peroxidation was assessed in liver homogenates through the measurement of malondialdehyde (MDA) formation using the thiobarbituric acid method [22]. Protein concentration was determined in accordance with the Bradford method by using bovine serum albumin as standard [23].

### 2.5. Histology Determination

Liver tissues were fixed in 4% paraformaldehyde in phosphate buffered saline (PBS). Subsequently, tissue samples were embedded in paraffin and 5 *μ*m thick sections were obtained. Sections were prepared for immunohistochemistry and hematoxylin and eosin (H&E) staining. All stained slides were visualized by using a light microscope (80i, Eclipse, Nikon®, Tokyo, Japan).

### 2.6. Immunohistochemistry Assay

Immunohistochemical (IHC) staining was performed by using an immunoperoxidase protocol. Sections were dewaxed overnight at 58°C. The specimens were hydrated in xylene (3 × 5 min) and 90% alcohol (4 × 3 min). Subsequently, sections were submerged in 1x PBS (3 × 5 min), autoclaved with 0.10 N citrate buffer at 121°C for 20 min, and washed again with 1x PBS (3 × 5 min). Subsequently, endogenous peroxidase was blocked with methanol peroxidase (46 mL of MeOH + 4 mL of H_2_O_2_) for 60 min, followed by five washes in 1x PBS for 5 min. Furthermore, to block nonspecific binding, 5% milk in 1x PBS was added for 60 min. The tissues were then rinsed with 1x PBS for 5 × 5 min. Subsequently, the tissues were incubated with primary antibody diluted in 3% fetal bovine serum overnight and rinsed with 1x PBS (5 × 5 min). The antibody used for IHC was targeted against nuclear factor kappaB (NF-*κ*B; p65) and is described in Table 2.

The tissues were incubated with secondary antibody for 2 h at 18–21°C (room temperature) and then rinsed with 1x PBS (5 × 5 min). Four hundred microliters of the peroxidase substrate 3,3′-diaminobenzidine ((DAB); 40 *μ*L DAB in 360 *μ*L H_2_O_2_) was added, incubated for 45 min, and rinsed with 1x PBS (5 × 5 min). Finally, the stains were counterstained with hematoxylin for 1 min and rinsed with 1x PBS (2 × 5 min), and the tissues were dehydrated in alcohol (4 × 80 s) and xylene (3 × 5 min).

The stained specimens were covered with resin and allowed to dry for 2 days. All stained slides were visualized by using a light microscope (80i, Eclipse, Nikon, Tokyo, Japan). Brown staining of proteins on the tissue was considered a positive reaction. Digital images of the histological sections were collected, and a positive signal was quantified by using ImageJ® software (NIH, MD, USA) [24].

### 2.7. Western Blotting Analysis

For protein analysis by Western blotting, the liver tissue was homogenized in lysis buffer (1 mol/L Tris-HCl pH 8, 5 mol/L NaCl, NP40, Triton, 0.5 mol/L EDTA pH 8, 0.1 mol/L PMSF, 0.1 mol/L Na_3_VO_4_, and 0.1 mol/L NaF [Sigma-Aldrich®, Missouri, USA] plus protease and phosphatase inhibitor cocktails [Sigma-Aldrich, Missouri, USA]) and then centrifuged at 12,000 rpm (13,200*g*) for 20 min at 4°C. The supernatant was recovered, and the protein concentration was measured by using the bicinchoninic acid method [25] (Pierce BCA Protein Assay catalog number 23223, Thermo Fischer Scientific®, NY, USA). Forty micrograms of protein per sample was separated by sodium dodecyl sulfate-polyacrylamide gel electrophoresis (SDS-PAGE) using a 12% gel (100 V, 3 h, room temperature); subsequently, the proteins were electrotransferred onto a 0.45 *μ*m immuno-Blot PVDF membrane (BIO-RAD®, CA, USA) (0.25 A and 1.40 h at 4°C). Nonspecific binding to the membranes was blocked by using 5% bovine serum albumin (BSA) (Sigma-Aldrich, Missouri, USA) in Tris-buffered saline with Tween-20 (TBST) for 2 h at room temperature. The membranes were incubated overnight at 4°C in a 500-fold diluted solution of primary antibodies, washed three times in TBST, and incubated for 2 h at room temperature in a 5000-fold diluted solution of secondary antibody. The primary antibodies against 4-hydroxynonenal (4-HNE), nuclear factor (erythroid-derived 2)-like 2 (Nrf2), tumor necrosis factor alpha (TNF-*α*), interleukin-(IL-) 1 beta (1*β*), p65, IL-6, and IL-10 are shown in Table 2. The antibody against *β*-actin was used as the internal control, and the results were expressed as a ratio relative to the control. The membranes were bathed in luminol reagent (Santa Cruz Biotechnology®, CA, USA) for development, and the photographic plates (catalog number 822526, Kodak®, NY, USA) were immersed for 5 min into the developer solution (catalog number 1900943, Kodak, NY, USA), rinsed with tap water, and placed in a container with fixative solution (catalog 1901875, Kodak, NY, USA) for 5 min. The plates were rinsed with tap water, allowed to dry, and imaged. The images were digitized, and the intensity of each band was quantified by using densitometric scanning with ImageJ software (NIH, MD, USA) [24].

### 2.8. Ribonucleic Acid (RNA) Extraction and Reverse Transcription Polymerase Chain Reaction (RT-PCR)

Liver samples (0.1 g) were immediately placed into cold TRI Reagent (Sigma-Aldrich, Missouri, USA) and frozen in liquid nitrogen. RNA was separated by using chloroform, isopropanol, and ethanol reagents, consecutively. The supernatant was centrifuged for 6 min at 10,600 rpm (11,500*g*) and resuspended in water. RNA concentration was determined spectrophotometrically (Nanodrop Lite, Thermo Fischer Scientific, Shanghai, China) at 260 nm. RNA was reverse transcribed in 19.3 *μ*L of reaction mixture, which consisted of 10 *μ*L of sample added to 2.5 *μ*L RT buffer, 1 U/*μ*L oligo (dT), 1.0 *μ*L RNAse inhibitor (40 U/*μ*L), 0.5 mM dNTP mix, and 0.3 *μ*L M-MuLV reverse transcriptase (200 U/*μ*L) (New England BioLabs, MA, USA). The following reverse transcription cycle was used: 65°C for 5 min, 37°C for 60 min, 70°C for 15 min, and 4°C for 5 min.

PCR was performed by using the Applied Biosystems® Step-One Plus Real-Time PCR system with software version 2.3. Taq polymerase (TaqMan, Universal Master Mix REF 4304437, Applied Biosystems, CA, USA) was used for the analysis of the relative expression of glutathione peroxidase (GPx). The tests were performed in duplicate and labeled with FAM reporter dye and a nonfluorescent quencher. The primers were purchased from Thermo Fisher Technology® (MA, USA). The reaction mixture totaled 10 *μ*L, containing 5 *μ*L of TaqMan Universal Master Mix, 0.5 *μ*L of GPx probe (Rn00577994_g1), 1 *μ*L of sample (16 ng/*μ*L), and 3.5 *μ*L of sterile H_2_O. The PCR cycling conditions were 95°C for 10 min and followed by 40 cycles of 95°C for 15 s and 60°C for 1 s. GAPDH (Rn01775763_g1) was used as the internal control, and the results were expressed as a ratio relative to the control. The baseline and threshold were set using the auto-baseline and threshold feature in StepOne Software (Applied Biosystems, CA, USA). The samples were considered positive if target amplification occurred within 35 cycles. A probe control, no template control (NTC), a target reagent sample, and a negative sample (without reverse transcriptase enzyme) were included on the plate. Standard curves were prepared by using a liver sample from the control group, which was serially diluted five times (1 : 0, 1 : 2, 1 : 4, 1 : 8, 1 : 16, and 1 : 32). A standard curve of the cycle threshold (C_T_) values versus the logarithm of the dilution was constructed, and the equation was calculated. The relative expression data were extrapolated from the standard curve.

### 2.9. Statistical Analyses

All graphical data were shown as the mean ± standard error (SE). Multiple comparisons were computed by using GraphPad Prism® 7.0 software (CA, USA). The results were analyzed by using one-way ANOVA between multiple groups, followed by Tukey's test. A *P* value less than 0.05 was considered significant.

## 3. Results

### 3.1. Stevia Prevents Acute Liver Damage

**Figure 1:**
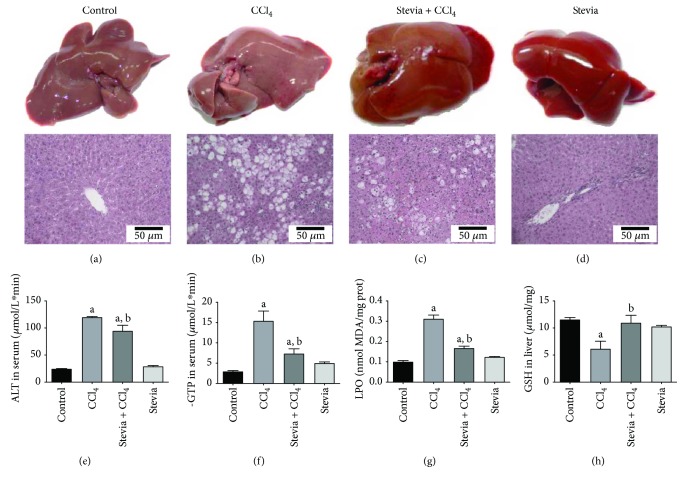
Stevia prevents acute liver injury. The effect of stevia on the macroscopic structure and on hematoxylin and eosin staining in the livers of control rats. (a) CCl_4_-treated rats; (b) CCl_4_ + stevia-treated rats; and (c) rats administered with stevia alone (d). Histograms depicting alanine aminotransferase (ALT) (e), gamma-glutamyl transpeptidase (*γ*-GTP) (f), serum activities, the degree of liver lipid peroxidation (LPO) (g), and the reduced glutathione (GSH) (h) content in acute CCl_4_-treated rats. Each bar represents the mean value of experiments performed in duplicate ± SE (*n* = 8). ^a^*P* < 0.05 versus the control group; ^b^*P* < 0.05 versus the CCl_4_ group.

As a pilot study to investigate whether stevia may be a potential treatment for liver diseases, we conducted an experiment using Wistar rats with acute liver damage induced by a high dose of CCl_4_. The general appearance of the livers at the macroscopic and microscopic levels is shown in Figure 1. Treatment with an acute dose of CCl_4_ produced inflammation of the liver that was prevented by stevia; the livers of stevia-treated rats were macroscopically similar to those of control animals. Hematoxylin and eosin-stained samples are shown in Figures 1(a)–1(d). The control group, depicted in Figure 1(a), showed an unaltered normal hepatic parenchyma. A representative section of the liver of acute CCl_4_-treated rats is shown in Figure 1(b); in this case, the tissue shows severe ballooning degeneration of hepatocytes (steatosis), hepatic parenchymal disruption, necrosis, and inflammatory infiltration. These alterations were attenuated by stevia (Figure 1(c)). Stevia treatment of control rats did not affect liver histology (Figure 1(d)).

The acute administration of CCl_4_ significantly increased the serum activity of ALT (Figure 1(e)), an indicator of hepatocyte necrosis [26], and *γ*-GTP (Figure 1(f)), a marker of cholestasis [26], relative to the levels in the control group. Stevia pretreatment completely prevented the increase in ALT and *γ*-GTP activities, which suggested that stevia can prevent necrosis and cholestasis. Stevia treatment of control rats did not affect the serum markers of liver damage.

MDA, one of the main products of lipid peroxidation, is commonly utilized to measure oxidative stress in tissues [27]. Acute CCl_4_ intoxication triggered lipid peroxidation, as indicated by MDA levels that were 3-fold higher than those in the control group. The antioxidant activity of stevia partially, but significantly, prevented the lipid peroxidation process (Figure 1(g)). Oxidative stress induced by CCl_4_ led to a reduction in the GSH concentration in the liver, which was also prevented by stevia (Figure 1(h)). Stevia treatment of control rats did not affect these oxidative stress parameters.

### 3.2. Stevia Prevents Chronic Liver Injury

**Figure 2:**
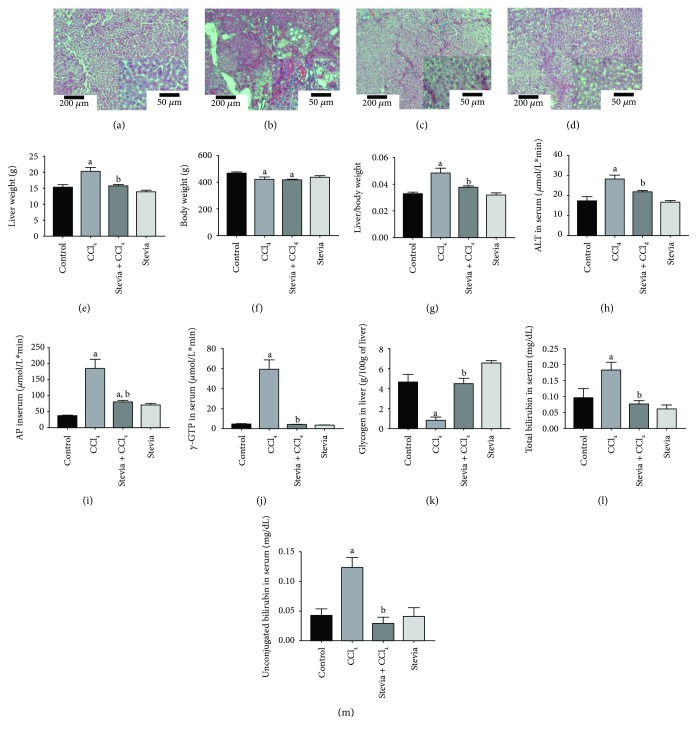
Stevia prevents chronic liver inflammation, necrosis, and cholestasis in chronic liver damage. The representative hematoxylin and eosin staining of liver sections obtained from the control (a), CCl_4_ (b), CCl_4_ + stevia (c), and stevia-treated rats (d). Histograms depict the liver (e), body (f), and body : liver weight ratio (g), alanine aminotransferase (ALT) (h), alkaline phosphatase (AP) (i), and gamma-glutamyl transpeptidase (*γ*-GTP) (j) serum activities, and liver glycogen content (k), total (l), and unconjugated serum bilirubin (m) levels. Each bar represents the mean value of experiments performed in duplicate ± SE (*n* = 8). ^a^*P* < 0.05 versus the control group; ^b^*P* < 0.05 versus the CCl_4_ group.

As we observed that stevia treatment effectively prevented acute liver damage, we decided to evaluate whether this plant was capable of preventing CCl_4_-induced cirrhosis. Hematoxylin and eosin staining of liver sections are shown in Figures 2(a)–2(d). The control group depicted in Figure 2(a) showed no alterations of the liver parenchyma. The image in Figure 2(b) corresponds to a representative hepatic section of chronic CCl_4_-induced liver damage; in this case, the tissue showed disruption of the liver parenchyma, steatosis, hyperchromatic nuclear hepatocytes, and atypical and pleomorphic nuclei. The alterations produced by CCl_4_ administration were prevented by stevia treatment (Figure 2(c)), but stevia treatment of control rats did not affect liver histology (Figure 2(d)).

The liver weight in the cirrhotic group increased compared with that in the control group, whereas stevia cotreatment prevented this increase (Figure 2(e)). The body weight was slightly decreased by chronic CCl_4_ intoxication, but stevia was incapable of maintaining body weight within control values (Figure 2(f)). The liver : body weight ratio was significantly increased by chronic CCl_4_ administration, although this effect was completely prevented by stevia (Figure 2(g)). Stevia treatment in control rats did not affect the liver, body, or liver : body weight ratio. The conventional serum markers of liver damage, ALT (Figure 2(h)), AP (Figure 2(i)), and *γ*-GTP (Figure 2(j)), were significantly augmented by CCl_4_ treatment. Cotreatment with stevia significantly prevented the increase of these serum indicators of hepatic injury. Glycogen, the main source of energy in the liver [28], and blood bilirubin were measured to assess the functional capacity of the organ. A dramatic decrease in liver glycogen content in the cirrhotic rats was observed compared with that in the control group (Figure 2(k)). Notably, stevia completely prevented this effect. The plasma concentration of total (Figure 2(l)) and unconjugated (Figure 2(m)) bilirubin was significantly increased by chronic CCl_4_ intoxication, whereas stevia treatment completely prevented this elevation. The administration of stevia in control rats did not affect glycogen or bilirubin levels.

### 3.3. Stevia Preserves the Redox Liver Balance in Chronic Liver Injury

**Figure 3:**
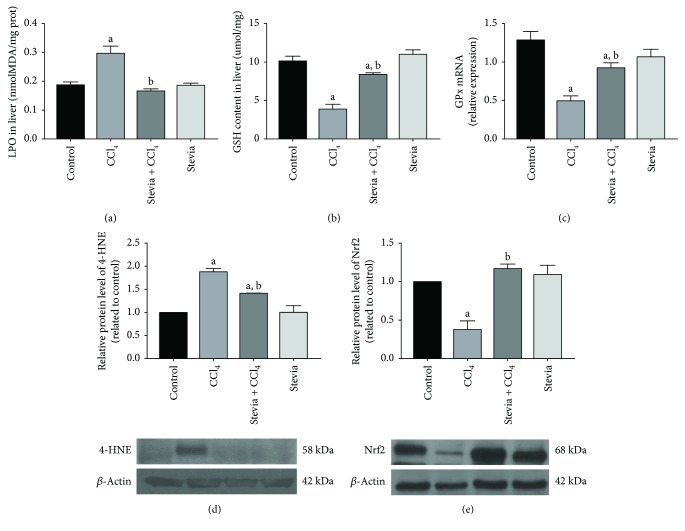
Stevia prevents oxidative damage in chronic liver damage. Liver lipid peroxidation (LPO) (a), reduced glutathione (GSH) (b), and glutathione peroxidase (GPx) (c) are shown. Each bar represents the mean value of experiments performed in duplicate ± SE. ^a^*P* < 0.05 versus the control group; ^b^*P* < 0.05 versus the CCl_4_ group (*n* = 8). Protein levels of 4-hydroxynonenal (4-HNE) (d) and nuclear factor (erythroid-derived 2)-like 2 (Nrf2) (e) in liver sections were determined by Western blot analysis of control, CCl_4_, CCl_4_ + stevia, and stevia-treated rats. Each bar represents the mean value of experiments performed in duplicate ± SE (*n* = 3). *β*-actin was used as a control. The values are expressed as the fold increase of OD normalized to the control group values (control = 1). ^a^*P* < 0.05 versus the control group; ^b^*P* < 0.05 versus the CCl_4_ group.

Oxidative stress plays a fundamental role in the development of liver cirrhosis [29, 30]. Five different indicators of oxidative stress were measured to investigate the antioxidant capacity of stevia. Lipid peroxidation and GSH are typical indicators of oxidative stress at the lipophilic and hydrophilic levels, respectively. In cirrhotic rats, lipid peroxidation increased (Figure 3(a)) and GSH levels decreased (Figure 3(b)) in the liver; importantly, stevia administration significantly prevented these alterations, demonstrating strong antioxidant activity. GPx is an important enzyme that acts against ROS, utilizing GSH to detoxify H_2_O_2_ [31]; this enzyme significantly decreased after CCl_4_ treatment, whereas stevia partially, but significantly, preserved GPx mRNA (Figure 3(c)). 4-HNE is a potent inducer of intracellular peroxide production and consequently exerts oxidative stress on cells [32]; as can be seen in Figure 3(d), chronic CCl_4_ treatment increased 4-HNE, which was significantly prevented by stevia. Nrf2 is considered a master regulator of the antioxidant response of the cell [33]; thus, we explored the possibility that, in addition to direct free radical scavenging activity of the antioxidants present in stevia leaves, this plant may induce the expression of Nrf2 as an important mechanism to modulate the redox state of hepatic cells. Cirrhotic rats exhibit a decreased level of Nrf2, although stevia completely preserved Nrf2 expression (Figure 3(e)). Stevia treatment of control rats did not affect these markers of oxidative stress.

### 3.4. Stevia Prevents Necrosis and Inflammation by Blocking NF-*κ*B and Proinflammatory Cytokines

**Figure 4:**
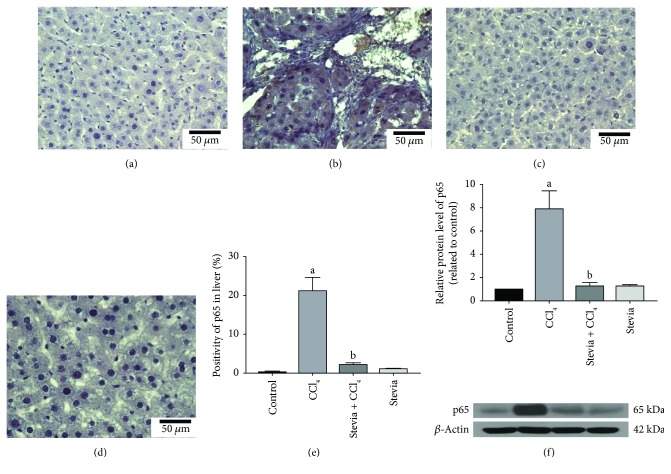
Stevia prevents the activation of the proinflammatory factor NF-*κ*B (p65) in chronic liver damage. Representative p65 immunohistochemistry of liver slices from control (a), CCl_4_- (b), CCl_4_ + stevia- (c), and stevia- (d) treated rats. (e) depicts the percentage of positivity of p65 obtained from immunohistochemistry slices. (f) depicts the protein levels in the samples of liver tissue determined by Western blot analysis. The values are expressed as the fold increase of OD normalized to the control group values (control = 1). Each bar represents the mean value of three rats ± SE (*n* = 3). ^a^*P* < 0.05 versus the control group; ^b^*P* < 0.05 versus the CCl_4_ group.

NF-*κ*B, the master regulator of inflammation and liver fibrosis, induces the expression of proinflammatory cytokines [34, 35]. As shown in Figure 4, liver tissues were embedded in paraffin and IHC with NF-*κ*B (p65) antibody was performed to investigate the effect of stevia on chronic liver damage. The figure demonstrates that few specific antigen detections of p65 were observed in the control group (Figure 4(a)). In contrast, the expression of p65 was significantly higher in the CCl_4_ group (Figure 4(b)) than in the control group. Notably, stevia cotreatment significantly ameliorated the induced upregulation of p65 (Figure 4(c)) relative to untreated cirrhotic animals.  Figure 4(e) shows that the percentage of positive zones was obtained from three liver slices (Figure 4(e)). The results were confirmed by Western blotting (Figure 4(f)), where it was observed that CCl_4_ induced an 8-fold increase in p65 protein expression, which was significantly attenuated by stevia treatment. Stevia treatment of control rats did not affect p65 expression.

**Figure 5:**
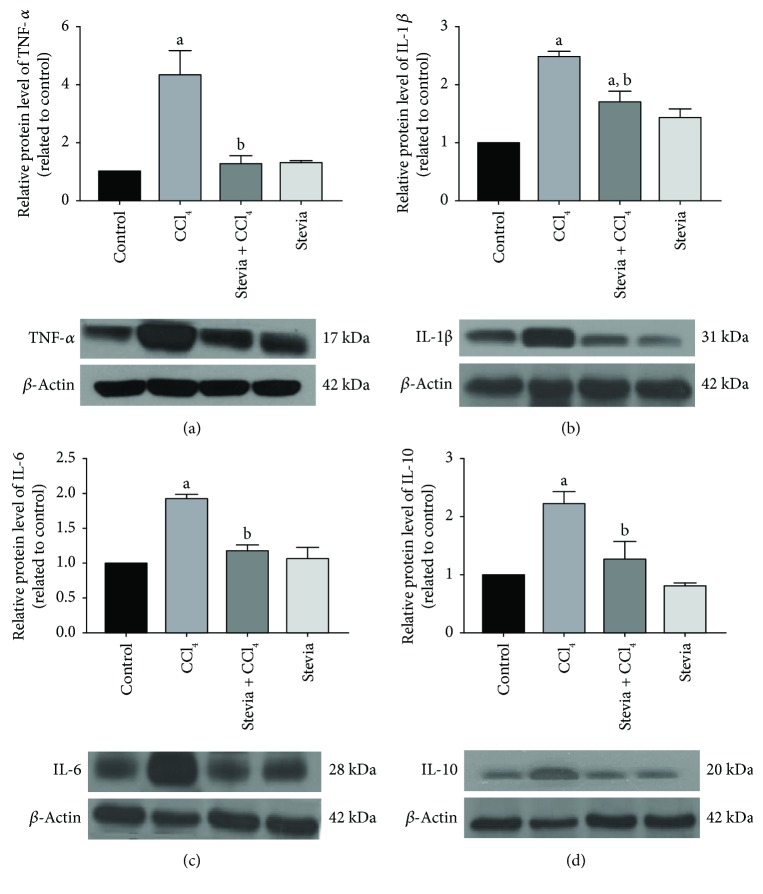
Stevia prevents the expression of proinflammatory cytokines in chronic liver damage. Protein levels of TNF-*α* (a), IL-1*β* (b), IL-6 (c), and IL-10 (d) in samples of liver tissue were determined by Western blot analysis of the control, CCl_4_-, CCl_4_ + stevia-, and stevia-treated rats. *β*-actin was used as a control. The values are expressed as the fold increase of OD normalized to the control group values (control = 1). Each bar represents the mean value of three rats ± SE (*n* = 3). ^a^*P* < 0.05 versus the control group; ^b^*P* < 0.05 versus the CCl_4_ group.

Several cytokines involved in inflammatory processes in liver damage are upregulated by NF-*κ*B, including TNF-*α*, IL-1*β*, IL-6, and IL-10 [36]. The levels of TNF-*α* (Figure 5(a)), IL-1*β* (Figure 5(b)), IL-6 (Figure 5(c)), and IL-10 (Figure 5(d)) were increased 4.1-, 2.5-, 2-, and 2.3-fold, respectively, in the livers of cirrhotic rats relative to the control group. Stevia treatment prevented the increased expression of these cytokines. These results demonstrate that stevia blocks the expression of NF-*κ*B proinflammatory signal transduction in CCl_4_-induced chronic liver injury. Stevia treatment of the control rats did not affect these cytokines.

## 4. Discussion

**Figure 6:**
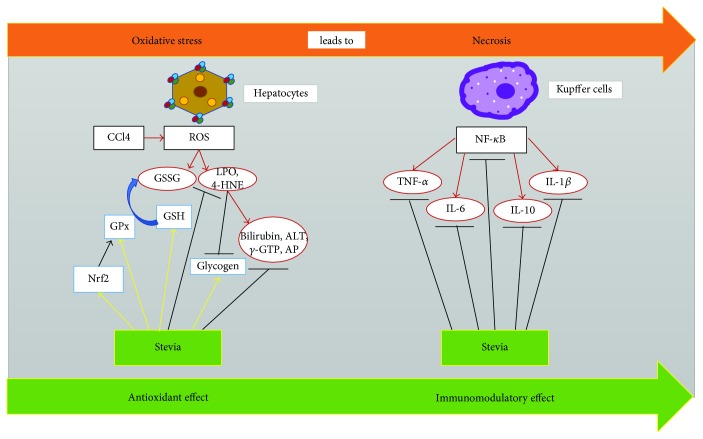
Schematic representation of the multitarget effect of stevia on experimental liver injury. Stevia may act at many steps to prevent liver damage. The wide spectrum of activities displayed by stevia can be summarized in three main categories: antioxidant, immunomodulatory, and antifibrotic effects. Antioxidant effects can be explained by direct free radical scavenging properties, increased Nrf2, antioxidant enzymes, and GSH. The immunomodulatory properties of stevia are associated with its ability to block NF-*κ*B and, subsequently, the proinflammatory cytokines TNF-*α*, IL-1*β*, and IL-6.

It is noticeable that the capacity of stevia leaves to improve the functional markers of acute liver damage is paralleled by its ability to prevent the changes in the markers of oxidative stress produced by acute CCl_4_ intoxication. In this regard, it is well known that free radicals play an important causative role in liver diseases [10, 29, 36–38]. These results prompted us to investigate the antioxidant and hepatoprotective properties of stevia on chronic liver damage produced by this hepatotoxic compound. Next, we evaluated the effect of stevia leaves in a liver cirrhosis model that shares several features with human disease [39]. We found that stevia prevents CCl_4_-induced liver cirrhosis in rats. Stevia preserved serum markers of necrosis (ALT) and cholestasis (AP, *γ*-GTP, and bilirubin), as well as the normal structure of the liver parenchyma. It also significantly ameliorated inflammation and maintained liver function in the storage of energy as glycogen. The protective mechanism of stevia was studied; it was found that the plant exhibits antioxidant and immunomodulatory properties by blocking or upregulating important molecular pathways that assist in the prevention of liver injury. The antioxidant effect of stevia in this cirrhosis model was shown to be associated with the ability of the plant to prevent the elevation of lipid peroxidation and 4-HNE, which are markers of oxidative stress in membranes [40], and to prevent the downregulation of liver GSH [41], an indicator of oxidative stress in the cytosol. Moreover, stevia prevented the decrease in Nrf2 and GPx observed in cirrhotic rats. The immunomodulatory effect was related to the ability of stevia to downregulate the proinflammatory factor NF-*κ*B [34] and, consequently, the harmful cytokines TNF-*α*, IL-1*β*, and IL-6. A graphical description of the multitarget protective effects of stevia is shown in Figure 6.

### 4.1. Involvement of the Nrf2 Signaling Pathway in the Hepatoprotective Activity of Stevia

It is well known that free radicals play a significant role in the establishment, development, and perpetuation of liver diseases [29, 36, 37]. ROS can generate toxic effects such as lipid peroxidation, enzyme inactivation, DNA mutation, and the destruction of cell membranes [42]. Thus, we investigated whether the protective effects of stevia were associated with antioxidant activity. A summary of the most abundant compounds present in stevia leaves is shown in Table 1. Some compounds, such as flavonoids, are known to possess important antioxidant properties [43, 44]; however, they constitute only a small percentage of the active components of stevia leaves (Table 1). Perhaps, the most abundant active components of stevia are stevioside and rebaudioside A, which possess only minor importance as free radical scavengers [2] as determined by the DPPH test (data not shown). In contrast, it is noteworthy that stevia treatment of CCl_4_-treated animals resulted in the prevention of oxidative markers such as MDA, GSH, GPx, and 4-HNE. Thus, these results prompted us to investigate whether stevia leaves could induce the expression of Nrf2, a key regulator of redox signaling in the liver [37, 38]. Chronic CCl_4_ treatment resulted in decreased expression of Nrf2 in the livers. Notably, we observed for the first time that stevia effectively prevented the downregulation of this antioxidant factor. Because the Nrf2 pathway is an endogenous cytoprotective system that may reduce levels of reactive metabolites through an effect on antioxidant enzyme expression [45], it is reasonable to speculate that preservation of Nrf2 levels by stevia could contribute to the antioxidant properties of the plant. In this way, stevia affords protection against liver damage by supporting the redox balance of the cell.

### 4.2. The Immunomodulatory Ability of Stevia Protects the Liver from Damage

NF-*κ*B is, undoubtedly, the orchestrator of the inflammatory response [34, 46]. p65 is one of the most prevalent members of the NF-*κ*B family and requires the activation of I*κ*B phosphorylation, its protein inhibitor, to further promote gene activation of IL-1, IL-6, and TNF-*α*; all of which are closely linked to inflammation [47]. Direct activation of NF-*κ*B through ROS is still controversial; however, the production of ROS upon IL-1*β* and TNF-*α* stimulation could lead to its activation [48]. The NF-*κ*B signaling pathway upregulates proinflammatory proteins such as TNF-*α*, IL-1*β*, and IL-6, which have been the focus of investigation of inflammatory organ injury because the uncontrolled and prolonged action of these proteins is potentially harmful [35]. Considerable evidence has accumulated to suggest that the NF-*κ*B signaling pathway contributes to the pathogenesis of liver inflammatory diseases through the activation of TNF-*α*, IL-6, and IL-1*β* [34, 35]. Moreover, patients with alcoholic hepatitis and cirrhosis showed elevated serum concentrations of these cytokines, including IL-6 and IL-8, where expression levels correlated with markers of liver function and clinical outcome [35, 49]. With respect to IL-10, its biological effect may be dependent on interaction with other cytokines [50]. We believe that the most likely mechanism of action of stevia in the downregulation of NF-*κ*B in chronic liver damage is antioxidation [2]. Therefore, the inhibition of NF-*κ*B by stevia leads to the downregulation of the proinflammatory cascade and, in turn, to the prevention of necrosis and cholestasis, and preservation of the liver parenchyma structure and function.

In conclusion, our results present the first demonstration that stevia prevents acute and chronic experimental liver damage in the rat [48] via a multitarget action. Stevia upregulates Nrf2 and therefore counteracts oxidative stress in the damaged liver. In addition, the plant prevents necrosis and cholestasis through the modulation of the main proinflammatory cytokines via inhibition of the NF-*κ*B pathway (Figure 6). Our current results present robust evidence of the immunomodulatory effect of stevia. In addition, stevia has a reasonable safety profile and is therefore suitable for clinical use. However, further studies are required in animal models to provide a rationale for the subsequent short- and long-term safety studies in patients with cirrhosis.

## Figures and Tables

**Table 1:** Main compounds of *Stevia rebaudiana* variety Morita II.

Compounds	Content
Carbohydrates	67.32%
Crude fiber	9.52%
Protein	12.11%
Fat	3.23%
Ash	7.82%
Chlorophyll	7 mg/g
Carotenoids	4 mg/g
Flavonoid compounds	36.7 mg quercetin equivalents/g
Phenolic content	28.4 mg gallic acid equivalents/g
Stevioside	15.5 g/100 g
Rebaudioside A	9.12 g/100 g

Data were obtained from references [2, 11–15].

**Table 2:** Antibodies used in Western blot and immunohistochemistry techniques.

Protein	Brand	Catalogue
4-HNA	Abcam® (Cambridge, UK)	AB46545
Nrf2	Abcam® (Cambridge, UK)	AB31163
p65	Merck Millipore® (MA, USA)	MAB3026
*β*-actin	Ambion® (MA, USA)	AM4302
IL-1*β*	Abcam® (Cambridge, UK)	AB18329
IL-6	Invitrogen® (CA, USA)	ARC0962
IL-10	Invitrogen® (CA, USA)	ARC 9102
TNF-*α*	eBioscience (CA, USA)	BMS175
